# “What’s in It for the Kid?”: An Approach for the Bedside Ethicist

**DOI:** 10.3390/children13050707

**Published:** 2026-05-21

**Authors:** Giuliana C. Antolovich, Ingrid Sutherland, Zoe McCallum, Monica S. Cooper

**Affiliations:** 1 Department of Neurodevelopment and Disability, The Royal Children’s Hospital, Melbourne, VIC 3052, Australia; giuliana.antolovich@rch.org.au (G.C.A.); ingrid.sutherland@rch.org.au (I.S.); zoe.mccallum@rch.org.au (Z.M.); 2 Department of Paediatrics, University of Melbourne, Melbourne, VIC 3052, Australia; 3 Neurodisability and Rehabilitation, Murdoch Children’s Research Institute, Melbourne, VIC 3052, Australia; 4 Department of Nursing, University of Melbourne, Melbourne, VIC 3052, Australia

**Keywords:** bioethics, decision-making, disability, severe neurological impairment, best interest, harm

## Abstract

**Highlights:**

**What are the main findings?**

- “What’s in it for the kid?” recentres decisions focusing on the child’s lived experience, rather than the goals or needs of decision makers.
- It acts as an accessible tool that complements existing frameworks by clarifying risks, benefits, and meaning for the child.

**What are the implications of the main findings?**

- The approach makes ethical decision-making more practical, accessible, and explicitly child focused.
- It promotes clearer weighing of benefit versus burden from the child’s perspective.

**Abstract:**

Background/Objectives: Advances in paediatric care have increased the survival of children with severe neurological impairment, often accompanied by complex disability, multimorbidity, and a substantial treatment burden. Determining whether interventions provide meaningful benefit to the child is ethically challenging, particularly when decision-making is shared between parents and clinicians, while the child has limited capacity to participate directly in decision-making. This paper examines the guiding question “what’s in it for the kid?” as a means of strengthening child-centred ethical deliberation alongside established frameworks. Methods: We undertook a conceptual bioethical analysis informed by clinical experience in an inner city tertiary public hospital. The analysis focuses on children with severe neurological impairment and medical complexity. The paper critically examines how the guiding question aligns with and extends key ethical constructs, including shared decision-making, the Zone of Parental Discretion, the Best Interests Standard, and care ethics. Clinical scenarios are used illustratively to demonstrate application in practice. Results: Existing ethical frameworks form an important foundational structure for complex decision-making. The question “what’s in it for the kid” translates ethical principles into a practical moral prompt that centres the child as the subject of decision-making. It facilitates clearer consideration of risks, benefits meaningful to the child and lived experience and helps to distinguish the child’s interests from those of parents and clinicians. Its simplicity enhances accessibility and supports consistent use in complex, high-stakes decisions. Conclusions: “What’s in it for the kid?” is a pragmatic and accessible ethical prompt that complements established frameworks by translating them into clinically usable practice. It promotes explicit, child-focused deliberation and supports a more transparent and child-centred evaluation of benefit and burden, particularly in contexts of uncertainty and medical complexity.

## 1. Introduction

“We need to be able to see the child in all her complexity and expand our understanding of who she is and what we owe her” [1].

Paediatric decision-making is shared within a triad of the child, the parents, and clinicians [2]. However, the idealized view of a triadic relationship poses a number of challenges in practice. In many situations, the infant, child, or adolescent is not able to be an active participant in decision-making; this might be a consequence of their age (e.g., neonates, infants, young children), developmental stage (with evolving competence and capacity), or circumstances (e.g., an adolescent intubated in an intensive care setting). In these cases, the parent has a greater influence in decision-making. Parents may act as proxy decision makers, obligating them to act exclusively in the child’s best interests. More often, however, the concept of parental autonomy better defines their role. Parental autonomy acknowledges that parents are best placed to make decisions for their child [3,4] and permits parents to consider the needs of the child, their own needs, and those of their other children in the decision-making process [5]. Parents are considered best placed to decide what the child might want and choose and are usually motivated by and have the capacity to do what is ‘best’ for that child [6]. However, what is ‘best’ may be less clear when there is prognostic uncertainty or when there is disagreement about limitations to potentially life-prolonging treatments between parents and clinicians. The primacy of parental autonomy may also risk the moral claims of the child [7]. The process of balancing the perspectives, values, knowledge, and expertise of parents and clinicians [3] can sometimes be overwhelming and may inadvertently diminish the view of the child’s wishes, needs, and hopes.

There are a number of reasons that this decision-making triad may be less feasible to implement in children with severe neurological impairment (SNI) [8], including the severity of their disability, the requirement for technological support, and an inability to express their own hopes and desires. Children with SNI live with complex disabilities and medical comorbidities and require care and support for every aspect of their daily care and activity [8]. Medical advances, new technologies, and changes in community expectations and access to information have influenced the survival and longevity of children with neurodevelopmental disabilities, particularly for those with SNI [9]. Innovations in paediatric care and technological dependence have the potential to enhance function, participation, and quality of life. At the same time, this medical progress can prolong life, but with an intensive and new level of care required at home [10]. These advances have also created an ‘unnatural’ trajectory, where survival is accompanied by unprecedented levels of multimorbidity and medical complexity [11].

Longevity and increasing therapeutic options create opportunity, but knowing whether interventions are helping or harming a child is clinically and ethically complex for everyone. Parents face a deep ambiguity: they have a responsibility to the child and also hold a primal imperative to preserve their child’s life. Parents are charged with assuming an increased responsibility in navigating decision-making, confronting a multitude of available options and decision time points. Prognostic uncertainty often sits uncomfortably alongside the promise of hope offered by technological intervention and escalation of care. In the early stages of a disease or evolving disability, parents (and clinicians) may need time and a ‘trial of therapy’ to address and respond to the prognostic uncertainty and understand what a ‘good life’ might be for their child [12]. There is a risk that this evolves into a therapeutic momentum of an unrelenting escalation of care. Fears, concerns about risk, and the desire to keep a child alive can unintentionally cloud judgment and risk eclipsing what might matter most to the child, potentially losing a clear view of the child in the process.

This challenge is heightened when the child has intellectual or communication disabilities and cannot actively participate in decision-making [13]. Yet, regardless of decisional capacity, every child has preferences and hopes [1,14]. It is essential to attend to what the child may choose, gain, or lose, while also navigating the often inevitable risks [15]. However, representing the child [13] and creating space to consider their hopes and what matters to them, while ensuring this is meaningfully included in decisions about their care, remains challenging to realize in practice. The drive to preserve life must be weighed against the ethical challenge that prolongation can, at times, negatively impact the child’s own interests and experiences. This tension places a heavy responsibility on clinicians to reflect critically on the burden placed on parents, while navigating the complex moral terrain faced by all involved.

In this paper, we examine how “what’s in it for the kid?” aligns with, extends, and at times challenges (1) shared decision-making models, (2) the Zone of Parental Discretion, and (3) the goods of childhood and the Best Interests Standard, and (4) we explore how it can be considered alongside care ethics and rights-based theory [16,17,18,19]. In this exploration, we will deliberately return to the question “what’s in it for the kid?”, as good paediatric care should always loop back to the child and their needs. Finally, we draw on clinical narratives, provided in the Supplementary Materials (Supplement S1), to illustrate the situation, the dilemma, and how the question “what’s in it for the kid?” operates in real-world contexts and demonstrate how it keeps the child at the centre of decision-making.

In this piece, we will use the following terms: “child” to describe both children and adolescents, “parent” to describe parents or caregivers, and “clinician” to describe any member of the clinical team—nurses, allied health clinicians, doctors, etc. While our experience, and therefore this paper, is shaped by our work with children with severe neurological impairment (SNI), the question “what’s in it for the kid?” is applicable across paediatrics.

## 2. “What’s in It for the Kid?”

This guiding question evolved in the context of bedside care, and the use of the word “kid” was instinctive. It has, however, remained with intention. The deliberate use of the word “kid” captures and focusses our attention on the significance of the voice of the child. “The child” is already at risk of being the object of our discussions when the focus is on the perspectives and values of a parent and clinician. The use of the word “kid” shifts us from the formality and abstraction of “the child” and supports a clear emphasis on the individual experience and purpose of the child, allowing them to be the subject rather than the object of our deliberations about their fate [20,21]. At the bedside, we often take this one step further, directly referencing the child by name, asking ourselves, other clinicians, and parents “what’s in it for Luke/Grace/Rosie?” (Supplement S1). Acknowledging the child as the subject of our deliberations results in the child and their experience becoming more real in our considerations.

We contend that asking “what’s in it for the kid?” functions as a moral prompt, centring the child’s subjectivity and compelling reflection on the ethical obligations of both clinicians and parents toward the child as an individual with intrinsic value, lived experiences, and moral significance. This framing keeps the child at the forefront of clinical and ethical deliberation and performs crucial ethical work by resisting the dominance of parental or clinician perspectives. “What’s in it for the kid?” complements and enriches established ethical frameworks and foundational standards by distilling complex deliberations, clarifying what is at stake, and reorienting attention towards the child’s lived experience. 

The question “what’s in it for the kid?” focuses decision-making on the child. It helps parents separate their need for their child’s survival from considerations of what the child may experience. By keeping attention on the child’s situation, it encourages clearer thinking about outcomes that matter directly to the child. This reduces the risk that parental needs or hopes are mistaken for, or prioritized over, the child’s interests. It can mark a pivotal moment, acknowledging the profound difficulty parents face in contemplating their child’s death, yet encouraging a shift towards the child’s lived experience (Table 1).

## 3. Shared Decision-Making

Decision-making for children with SNI is never just about the medical condition of the child; the context of their family and community and the perspective of the doctor are also important influencing factors [22,23,24]. These experiences and perspectives have the potential to shape communication and decision-making in important ways. Shared decision-making (SDM) in paediatrics is a complex space with multiple stakeholders, diverse perspectives, and competing interests [18]. Although widely accepted as a core feature of paediatric practice, SDM is shaped by tensions between parental authority, clinician obligations, and the responsibility of both parties to make evidence-based decisions in the child’s best interests [18]. The process is influenced by barriers such as unequal knowledge, communication difficulties, and conflicting beliefs. There are also recognized facilitators such as trust, transparency, and ethical guidance [25,26]. As a collaborative model, SDM aspires to family-centred care, and compromise is often required [18].

Parental needs and concerns about the possibility of their child’s death are often at the forefront. Most parents of children with SNI will have already had to make many difficult decisions, often from soon after birth and sometimes prenatally [4,27,28]. Decisions about the introduction of technology to support respiratory function or nutrition, surgical interventions, and the trial of a new therapies have often been discussed. Many families have faced their child’s fragility, risk of early death, and repeated discussions about the direction of care and resuscitation, experiences that accumulate and frame parental perspectives [29,30,31]. Parents have reportedly found decisions burdensome, challenging [32], and traumatic, and are often left feeling like “every decision feels like an impossible choice” [33]. Parents are nevertheless accustomed to making these decisions and are positioned as best placed to do so [4]. Parents also use online communities to access medical information and guide their decisions. Peer advice is a powerful and recognized influence in consultations [34]. The chorus of voices, beliefs, ideals and opinions can crowd the decision-making space, which, in turn, can further remove the focus from the child themselves.

Clinical uncertainty, contested outcomes, and diverse values and beliefs can overwhelm the decision-making process [4,28,35]. Disagreement between parents and clinicians can intensify these tensions, potentially blurring the needs of the child [33]. The result is a dynamic where the child and their perspective and needs are repeatedly pushed to the side, albeit unintentionally.

Clinicians bring experience of supporting children and their families through complex decision-making processes. However, power imbalances between parents and clinicians complicate SDM [28]. Clinicians’ risk aversion and previous experiences and biases add additional strain. Clinicians have their own personal values [22], including beliefs about what is a good life [24]. Beliefs, values, assumptions, and risk aversion can influence the options offered, the communication of information, and the description of prognosis [36,37]. The successful application of “what’s in it for the kid?” requires the clinician to deeply understand their own values, beliefs, perspective, and biases and their potential impact [22,24]. It is not enough to ask the question “what’s in it for the kid?”, as without reflective practice, the clinician remains at risk of inserting their own beliefs, further threatening the outcome for the child. Reflective practice is supported by engaging in both informal conversations with colleagues and formal ethical process and practice, such as consultation with an ethics service. Accessing facilitated clinical supervision to interrogate and understand motivators and biases is another important opportunity to develop reflective practice.

In paediatrics, our shared decision-making centres on the interaction between clinician and parent with regard to the child. The child is positioned as the object of deliberation rather than the subject of decision-making, with all perspectives mediated through adults. For children with severe neurological impairment, the concern of limited future agency and autonomy means they may not move from object to subject within this process over time. The values and concerns of parents and clinicians may repeatedly override what the child actually wants [4]. While shared decision-making is foundational, it has gaps in high-stakes decisions for children with disabilities, particularly where decisional capacity is limited and may not evolve. A central concern is that the child has fewer opportunities to actively participate or be adequately represented. Communication and cognitive disabilities can pose significant barriers to healthcare decision-making for children with complex disabilities. This is particularly the case when decisions are more complex, when accommodations to support participation are difficult to implement, or, at worst, when there is a failure to even try to consider this need [21,37,38].

## 4. Zone of Parental Discretion

Parents are usually best placed to make decisions for their children, with their values and perspectives being central to defining their child’s best interests. The Zone of Parental Discretion (ZPD) permits parents to make decisions that may fall short of being medically ideal (i.e., not consistent with the treatment recommended by the medical team), provided they do not cause significant harm [16]. It recognizes parental authority while balancing this with the child’s best interests by accepting decisions that are ‘good enough’ [39].

Parents of children with disabilities provide lifelong care and support. Given the emotional, financial, and social weight of these decisions, parents of children with SNI frequently make more complex choices than those less familiar with healthcare. They may also be granted broader discretion to choose options that fit their capacities and family context and values.

In healthcare, the ZPD is used to navigate appropriate treatment when uncertainty or disagreement arises. While parents’ views are respected, professionals may intervene when choices clearly harm the child or neglect their best interests. The intention is always to promote the child’s overall wellbeing. In some circumstances, not overriding parental decisions contributes to overall wellbeing. While a single suboptimal decision may have no measurable consequence, the impact be of multiple and potentially sub-optimal decisions may result in cumulative harm. Can each tolerated decision subtly and slowly shift the boundary of what counts as acceptable risk and of what is regarded as optimal care for both the family and the clinical team? The question “what’s in it for the kid?” can help flag these smaller decisions that may not seem harmful in isolation but become damaging in accumulation.

## 5. Goods of Childhood and Best Interest Standard

**Figure 1:**
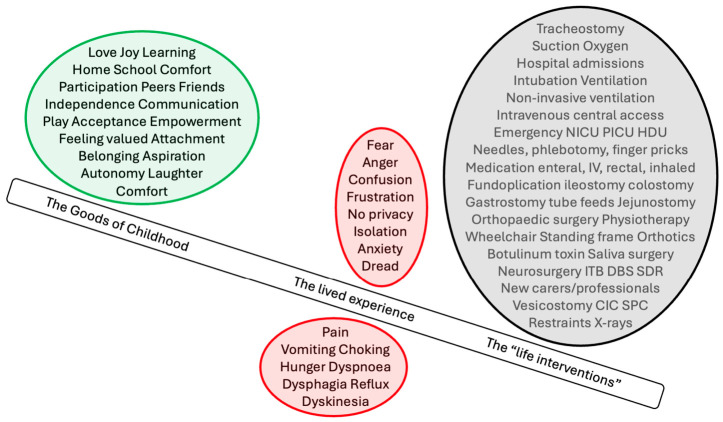
Reality of life with severe neurological impairment versus the goods of childhood: the lived experience of the actions, interventions, and emotional burden required for survival outweighs the idealized conception of childhood goods.

Alan Marshall’s I Can Jump Puddles [40] evokes the “goods of childhood,” portraying resilience, imagination, and joy despite disability. It reflects the life of a child with a disability but without a cognitive disability or a profound physical disability and risks idealizing childhood in ways that fail to capture the realities of a child with SNI (Figure 1, Table 2). Yet both children with and without a disability experience joy, love, and connection, though in different forms. For children with SNI, the goods of childhood are more relational, sensory, and care-based, while for typically developing children, they may also include autonomy, play, and cognitive exploration. These “goods” are not only sources of enjoyment but are essential for children to flourish and for their overall wellbeing during their formative years. For adult development, early experiences provide the groundwork for later autonomy, resilience, and emotional maturity. In relation to children’s rights, recognizing these goods affirms their entitlement to conditions that support both their present wellbeing and future potential. Importantly, the value of childhood is not diminished by impairment, but rather it is realized differently (Table 2).

Against this literary backdrop, the Best Interest Standard (BIS) has become part of clinicians’ daily lexicon and provides a valuable framework for care and decision-making. The BIS has emerged as the dominant framework in medicine, law, and child welfare when children cannot decide for themselves, directing attention to health, wellbeing, long-term outcomes, pain, suffering, and, where possible, the child’s views [7,41,42]. A potential constraint of the BIS when used in the context of children with SNI is the challenge of imagining a future with self-determination. In this context, best interests may instead be better focused on by the present and defined by freedom from pain, meaningful relationships, psychological wellbeing, schooling, social participation, and avoidance of repeated hospitalization. Daily life and circumstances may not reflect theoretical ideals or align with a described list of “best interests” [43]. While firmly embedded in clinical practice, the BIS is criticized as rigid yet vague, with scope for reasonable disagreement and ultimately conflict between parents and clinicians [44,45]. The BIS, while elegant in theory, often feels detached from the realities clinicians confront at the bedside and the experience of children with SNI (Figure 1). Both clinicians and parents may struggle to connect with abstract language when what is needed is plain, grounded conversation. Parents and clinicians must weigh the child’s interests, share information, explain their preferences, and respect the perspectives of others. Asking “what’s in it for the kid?” offers a way of operationalizing the BIS, directing attention to the child’s situation, lived experience, and needs. 

## 6. Care Ethics and a Rights-Based Approach

Care ethics locates moral responsibility within the relationships and lived realities of children. This is especially important for children with SNI, who depend on complex networks of care. A care ethics approach directs attention to lived experience, dependence, and the emotional realities of caregiving. It underscores the importance of responsiveness, empathy, and the moral weight carried within caregiver–child relationships, even when those relationships are unequal. A rights-based perspective complements this by affirming the child’s moral and legal status as a person with agency, entitlements, and dignity. Together, these frameworks resist reductive or paternalistic decision-making and require that children’s interests, preferences, and harms or benefits be meaningfully considered, even when they cannot be articulated by the children themselves. They also remind us that efforts to preserve life, however well-intentioned, can inadvertently marginalize the child’s own interests and experiences, and that parents shoulder this responsibility within an inherently asymmetrical role of authority and care. For children with SNI and high care needs, their daily dependence on carers and technology often requires a balance between burdens and compensatory goods, a balance that is not always achieved (Figure 1). Over time, routines of care can become normalized, and their cumulative impact on the child’s lived experience may be overlooked. Framed in simple terms, “what’s in it for the kid?” forces a pause to consider how to best represent the child and support their agency, capturing these commitments in practical terms. It operationalizes care ethics by directing both parents and clinicians to deliberate in ways that remain anchored in the child’s welfare and lived experience [12].

“Otherness” in disability arises when difference is constructed as deviation from the norm rather than recognized as part of human diversity [46]. When, disability is reduced to a category of lack, denying subjectivity and reducing individuals to abstraction, [47] the child risks being perceived primarily as an object of care. They are not recognized as moral subjects in their own right but are instead understood through the perspectives of others within clinical practice and parental identity.

## 7. Conclusions

Asking the question “what’s in it for the kid?” offers a way to ground decision-making for children. It directs ethical attention towards the child, keeping them at the centre of deliberation. The question helps distil what is truly at stake by considering what the child, rather than only the clinician or parent, might gain or lose. In this sense, it makes the Best Interests Standard tangible by amplifying the child’s needs, preferences, and lived experience. Positioned alongside shared decision-making, the Zone of Parental Discretion, and care ethics, this framing provides an additional tool to support the complex decisions in the care of children, particularly those with disabilities and high care needs. We propose that the question “what’s in it for the kid?” could serve as a moral prompt across hospital and community settings—for example, daily clinic or inpatient care (ward rounds, clinic appointments, multidisciplinary meetings, Advance Care Planning, and formal clinical ethics consultations.

This approach is singularly relevant as clinicians face increasingly challenging decisions in a rapidly evolving landscape of medical advances and treatment options. While developed in the context of children with SNI, this framing also address challenges inherent to many paediatric decision-making contexts, including elective procedures and even blood tests often considered “routine”. In this way, “what’s in it for the kid?” represents a practical, ethically grounded tool that keeps the child’s interests central across diverse clinical contexts.

## Figures and Tables

**Table 1:** The ethical “nuts and bolts”, the work and principles underpinning the “what’s in it for the kid?” approach. Developed from the authors’ clinical experience and conceptual analysis.

**Centres the child**
Focused consideration of the child’s experience, not others
Child at the heart of the decision-making
Child is the subject, not the object of discussion
**Distils the question to consider what is really at stake** **Puts clinicians and parents in “child’s shoes” and opens discussion to examine and discuss:**
What is meaningful to the child?
What brings them joy?
What does it mean to the child right now?
What might the decision mean for the future child?
What is the cost to the child?
**Enables overt examination of risks versus benefits to the child**
Focus on needs and potential gains of the child (not those of parent or clinician)
Helps us to consider risks in view of what the child might gain
Is there an acceptable balance between risks and benefits?
**Quietens “the chorus**”
Elevates the child’s “voice” (personhood)
Quietens the voices that are speaking to their own values, fears, and prior experiences
**Challenges us to understand how we might “best” represent the child**
**Examines parental and clinicians’ values, beliefs, hopes and desires:**
How are these influencing the discussion?
How are these influencing decisions?
How do they consider and incorporate what the child might want?
**Explores the risk that the values, beliefs, hopes, desires, and fears of the parent and the clinician may override what the child might want, or what “the child might choose for themselves”**
Encourages thinking about the gains, not only the risks
Requires reflexivity in practice
Critical when uncertainty prevails
**Questions whether the values and fears of the clinician influence the delivery of the message or the options presented**

**Table 2:** Reframing the Best Interest Standard: from typically developing children to those with severe neurological impairment.

Domain	Typically Developing Children	Children with Severe Neurological Impairment
Intrinsic Value of Life 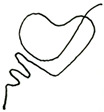	Life is valuable, often seen as a “a given” and understood in terms of potential for an open future Intrinsic value of childhood—not just a pathway to adulthood	Life is inherently valuable, and may be under threat of premature death Are the interventions required to sustain life tolerable for the child? Life at all costs?
Relationships and Emotional Connection 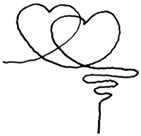	Communication, play, and emotional reciprocity to form attachment and complex relationships with family and peers	Responsive caregiving fosters a bond through voice, touch, eye contact, gesture, body language and movement, and presence and predictability Bonds with caregivers, siblings, and others which promote a sense of security and love and belonging Engaged with a broader circle of caregivers given medical complexity and therapies Establishment and maintenance of peer relationships complicated by barriers: physical, communication, environmental, opportunity, societal
Pleasure and Sensory Enjoyment 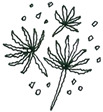	Pleasure from active, autonomous exploration, play, and social interaction	Enjoyment from tactile, auditory, and visual experiences (e.g., music, touch, nature) Comfort from familiar routines and environments, addressing sensory needs and positioning Reliant upon others to enable these experiences—to bring them to the child, to ensure the child is able and supported to participate
Security and Protection 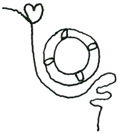	Security enables confident exploration Autonomy evolves As child grows older and more autonomous, safety increases, and they are able to provide their own care and communicate needs	Requires consistent and responsive caregiving Physical and emotional security Requires trust in systems, known communication methods Child needs to know that their needs will be anticipated, heard, understood, and responded to Need for environmental security—a stable home with modifications to enable full participation, such as an accessible bathroom or mobile hoist
Being Valued and Recognized, Belonging 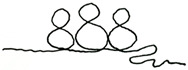	Achievement, participation, freedom of self-expression	Being treated with dignity Participation in family life, inclusion in daily routines, community participation, reinforce belonging with structures in place to ensure participation (access and carer support)—meaningful inclusion Awareness in community of disability needs Being visible in community through inclusion and representation: “You can’t be what you can’t see”
Education 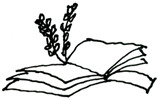	Fosters individual growth by developing critical thinking, communication, and problem-solving skills, while also preparing individuals to be autonomous, active, contributing members of society by imparting knowledge and interacting with others	A school environment that is adapted to ensure safe and enjoyable participation, learning, personal empowerment, and development of autonomy Critical importance of being able to express needs and wants—communication in both verbal and non-verbal means and with support of augmentative and alternative communication (AAC) Developmental level may not be reflected by chronological age in children with an intellectual disability; access to individual learning plan tailored to the child’s abilities and needs Children with a severe physical disability will require adapted teaching and learning environments to enable optimal participation and learning, e.g., specialized exercise equipment
Play and Leisure Adapted to Ability 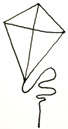	Active play, imagination, rules, and peer interaction	Engagement with sounds, movements, or interaction suited to functional, sensory, and developmental level Joy and stimulation through therapeutic or recreational activities Adapted activities, e.g., sensory play, passive movement Access to opportunities for play and participation through adapted programs and support for care givers (physical and financial)
Freedom from Pain and Distress 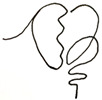	Focus on emotional regulation and learning to manage discomfort or frustration	Optimal medical care focused on detection of pain and emphasis on comfort and symptom management Quality of life Prevention of pain Secure and supported caregiver and home environment
Expression of Preferences 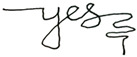	Multiple ways to communicate through language, communication, thoughts, choices, and emotions	Opportunities to express likes/dislikes and preferences Recognition of agency, even in limited forms
Spiritual and Existential Fulfilment 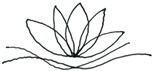	Involved through family-led rituals or presence in community traditions Emerges to form personal understanding and participation in cultural/spiritual life	Involved through family-led rituals or presence in community traditions Exposure to spiritual or cultural practices meaningful to the family and child Recognition of the child’s role within a broader community or belief system Complexity of cross-cultural perceptions of disability

## Data Availability

No new data were created to write this essay.

## Supplementary Materials

The following supporting information can be downloaded at: https://www.mdpi.com/article/10.3390/children13050707/s1, Supplementary Materials (Supplement S1).

## Abbreviations

The following abbreviations are used in this manuscript:

SNI	Severe neurological impairment
SDM	Shared decision-making
ZPD	Zone of Parental Discretion
BIS	Best Interests Standard
