# Supplementary material for: “What’s in It for the Kid?”: An Approach for the Bedside Ethicist
Source: Children (Basel). 2026 May 21;13(5):707. doi: 10.3390/children13050707 (PMC13204440; doi:10.3390/children13050707)
Supplement: Supplementary file 1 [file children-13-00707-s001.zip › children-4306367-supplementary.pdf]

Supplement S1 - Supplementary material

The clinical narratives. Luke, Grace, and Rosie's stories are an amalgam, drawing on several different children and families cared for by the authors. These narratives allow exploration of the application of "what's in it for the kid?". In each clinical story there are many competing needs, hopes, and wishes. Balancing those needs can be challenging. Posing the question "what's in it for the kid?" centres and amplifies the child's needs and focuses our ethical attention to what is really at stake.

Abbreviations used include:

Cerebral palsy (CP)
Gastroesophageal reflux disease (GORD)
Non-invasive ventilation (NIV)
Paediatric intensive care unit (PICU)
Traumatic brain injury (TBI)

Luke's story

Luke is 17 years-old, he is quadriplegic and wheelchair reliant following a traumatic brain injury (TBI) four years ago. He also has a severe cognitive and communication disability – he has no access to augmented communication support. Luke is fed by gastrostomy tube and requires carer support for all his daily activities. Luke developed respiratory failure following a minor procedure and now requires non-invasive ventilation (NIV) at night.

Luke has scoliosis – his spine is getting more curved and stiffer. He has increasing pain - he can only spend an hour in his wheelchair without becoming distressed. Luke loves going to school, and watching his younger brothers play sport, but his ability to do these activities is limited by his pain and his inability to spend time in his wheelchair.

Corrective scoliosis surgery is being considered. Laura, his mother, acknowledges Luke's severe disability that will shorten his life - she has prepared an Advanced Care Plan for him. Laura is very worried about surgery. The clinical team are also worried about his medical frailty and there is some disagreement about whether to recommend surgery for Luke.

**The situation**	**The dilemma**	**"What's in it for Luke?"**
Scoliosis correction surgery carries a high risk for Luke - a previous simple short procedure uncovered his poor respiratory health and his care needs have increased.<br><br>His mother Laura is very worried about the surgery. She knows him best and saw what he experienced after his original TBI - his suffering and how much his life changed. Laura has been clear that she does not want to increase Luke's "injury burden".	It is clear that Luke enjoys many things, and his younger brothers are important to him. He loves watching his brothers play sport and this is also a way for him to be part of the community. But Luke's life has contracted, his increasing pain limits his ability to leave the house and be part of his family's life. Will the surgery allow him to sit more comfortably in his chair and improve his participation? Are there other ways we can help him to manage his pain and improve his community access? The surgery is not without risk, and he is now established on NIV - is this an additional risk or will it be protective? Luke will need to be cared for in the paediatric intensive care unit (PICU) after the surgery, and the recovery might be prolonged.<br><br>But what will Luke gain? His respiratory function may be stabilised and slow his deterioration - this is an important consideration for the clinical team, but perhaps less so for Luke. Reducing his pain is a key goal for everyone involved. And for Luke, the key gain will be a chance to do the things he enjoys.<br><br>Will this help mitigate or balance the potential risks of the surgery for his mother and the clinical team? What might Luke do if he could choose?	Posing the question takes us closer to the key issues that might be relevant to Luke. For Luke, quality of life might be best measured by his participation in family and community life. Surgery may offer him the chance to be able to sit in his wheelchair and be "pain free". Luke's future is uncertain as he is living with a life limiting condition.<br><br>Does the potential for an improvement in his quality of life help us to balance the concerns about the risk of surgery? By considering Luke's needs and what he will gain, we can better balance the risks of the surgery itself, his mother's trauma, his mother's fears, and the clinical team's concern.

Grace's story

Grace is an eleven-year-old girl with severe cerebral palsy (CP). Grace is a wheelchair user and has a significant movement disorder, dystonia - she has an implanted neurosurgical device to help manage this problem. Grace has a profound intellectual and communication disability. Grace has many of the complications of CP - complex epilepsy; an unsafe swallow and gastrostomy tube feeds; and musculoskeletal disease. Grace also has severe gastroesophageal reflux disease (GORD) - she vomits frequently, and it is often blood stained. Grace has had several admissions with respiratory illness, some associated with aspiration because of her frequent vomiting. Grace has been very unwell in the last year and has been admitted to the PICU three times in the last 12 months. Grace requires NIV and supplemental oxygen when she is asleep. When she is at home and well, she goes to school every day.

Grace lives in a rural area, about two hours away from the city, and has a local care team that knows her very well.

Grace's dystonia is getting worse, and when it is bad, she can't control the movements of her mouth, jaw and neck. This often leads to vomiting and sometimes, aspiration. Her family feel pain is driving her dystonia. Grace has multiple *potential* contributors to her pain - GORD, dystonia and she has a dislocated right hip and severe progressive scoliosis.

Grace is being considered for surgical management for her dislocated hip and scoliosis. Her parents are advocating for the surgery, as they believe it will improve her pain and quality of life. However, the clinical team who know her well, feel she is too fragile to survive the surgery.

**The situation**	**The dilemma**	**"What's in it for Grace?"**
There is tension between the views of those who know Grace well - her family and the local clinical team.<br><br>Grace's parents are vigorous advocates, and they want to be sure she does not miss any opportunity. The clinical team - the paediatrician who has known her from birth and the nurses who provide both hospital based and home-based care - are very worried about the surgery. They have seen other children like Grace do very badly, and they worry she will end up in PICU again and need more and more interventions.	Grace's symptoms are significant. The dystonia and posturing of her jaw and mouth are very debilitating - a substantial source of pain and trigger vomiting, creating a risk of aspiration. Grace's parents are very worried about her pain. They have also quietly voiced that they are worried that Grace is not being considered for surgery because she has a disability.<br><br>The clinical team are worried that her pain and the terrible dystonia of her jaw and mouth are being driven by her severe GORD, *not* her musculoskeletal problems. The clinical team are very worried that even with surgery, these symptoms will not improve. There is also concern that the surgery might make things worse. They are even more worried that she won't survive the surgery.	Posing the question helps us focus on Grace. The most meaningful outcome for Grace is better management of her pain and dystonia.<br><br>However, it is very unlikely that surgery will improve either and therefore will not change the things that make Grace's life bad. Holding this truth for Grace will support both Grace and her family in this complex decision making.

Rosie’s story

Rosie is seven years old and has a rare and progressive genetic condition that has resulted in serious cardiac, respiratory and musculoskeletal problems. Rosie has a severe scoliosis - she is in pain every day and increasingly short of breath. Rosie is only able to walk very short distances, and this has limited her ability to do things that are meaningful to her - Rosie loves school is rarely able to go and is less able to do things with her three sisters.

Rosie is being considered for scoliosis correction surgery. There are a number of risks - she is young; her bones are not mature, making the surgery difficult; she has a serious cardiac condition; and very poor respiratory function.

The clinical team have concerns about doing surgery on her hips or spine. There is deep uncertainty about whether it would improve Rosie’s ability to walk and do things with her family. The clinical team are also concerned about how she would manage the surgery. It is likely that she will need a prolonged period of respiratory support in PICU after the surgery and there is a real concern she may need a tracheostomy. This will mean a long admission, time away from her family and home and potentially a very different burden of care for Rosie and her family when she does get home.

Rosie can tell us that her life is “not good”, and her parents agree. Rosie can’t do things that are important to her. Her parents have noticed that Rosie is sad and is more withdrawn at home. Her family want to consider surgery - they describe Rosie’s life as being “so diminished” and describe it is important to “take a chance” for her. They know she will have a short life, and they want it to be good.

The clinical team are divided - some think the surgery is worth the risk for Rosie’s quality of life, others very worried about what Rosie will go through if she has surgery.

**The situation**	**The dilemma**	**“What’s in it for Rosie?”**
Rosie has been able to articulate what she wants, well at least what she doesn’t like. She has also become withdrawn and quiet - is this because of pain, or fear, or because she feels disconnected from her family and her school community? The curve in her spine is making walking very difficult and increasingly effortful, compounding the direct impact of her scoliosis on her respiratory function.<br><br>Correcting her scoliosis will allow her to be more upright and this *may* improve her walking and endurance, but it may not make less breathless and will continue have poor respiratory health and more and more interventions.	Rosie’s goal is to be able to go to school and do more things with her sisters. Her parents are aware of her life limiting condition, and the potential risks of the surgery and recovery.<br><br>However, it is not clear if Rosie understands the seriousness of her condition, how long she might be away from home, or what having a tracheostomy might mean. Could this be a further barrier to her doing things with her sisters, or going to school?	Posing the question allows us to focus on Rosie’s needs and hopes. It allows us to create a space to gently explore each of these issues with Rosie and her family.<br><br>The clinical team are very focussed on avoiding risk - posing this question “What’s in it for the kid?” also helps the clinical team shift their focus away from just the risk and understand what Rosie might gain, help them see Rosie and the situation she is in.
